# MetaTreeMap: An Alternative Visualization Method for Displaying Metagenomic Phylogenic Trees

**DOI:** 10.1371/journal.pone.0158261

**Published:** 2016-06-23

**Authors:** Maxime Hebrard, Todd D. Taylor

**Affiliations:** Laboratory for Integrated Bioinformatics, Center for Integrative Medical Sciences, RIKEN, Yokohama, Kanagawa, Japan; Hospital for Sick Children, CANADA

## Abstract

Metagenomic samples can contain hundreds or thousands of different species. The most common method to identify these species is to sequence the samples and then classify the reads to nodes along a phylogenic tree. Linear representations of trees with so many nodes face legibility issues. In addition, such views are not optimal for appreciating the read quantity assigned to each node. The problem is exaggerated when comparison between multiple samples is needed. MetaTreeMap adapts a visualization method that addresses these weaknesses. The tree is represented by nested rectangles that illustrate the number or percentage of assigned reads. MetaTreeMap implements various options specific to phylogenic trees that allow for quick overview and investigation of the information. More generally, the goal of this software is to provide the user with the ability to easily display phylogenic trees based on various quantities assigned to the nodes, such as read number, percentage or other values. The tool can be used online at http://metasystems.riken.jp/visualization/treemap/.

## Introduction

In metagenomic studies samples usually consist of a mix of hundreds, or even thousands, of different species. In most cases, these species cannot be isolated or cultivated under laboratory conditions. Therefore, the entire sample is sequenced at once, then various methods such as MetaBin [1] have been developed to compare the sequences to known species and to assign the reads to specific taxa. Regardless of the methodology, the result of the taxonomic assignment is a phylogenic tree with a number of reads linked to each node (taxon). While trees with only a few dozen nodes are easy to display, large metagenomic trees contain so many taxa that they are difficult to visualize and comprehend. Not only do we need to legibly display the hierarchy of the tree, but we also need to know the number (and percentage) of reads assigned to each node, especially for comparing two or more samples. While various programs can display phylogenic trees, most of them, such as TreeDyn [2] and Dendroscope [3], focus on tree topology or branch length. And only a few programs, such as Krona [4], can handle a quantity (weight) assigned to nodes. Linear representation of large trees is cumbersome and node weights are almost meaningless. Circular representation succeeds to display trees in a smaller space, but labels displayed in multiple angles are difficult to read, and quantities remain difficult to appreciate and compare. To tackle these problems, we present an alternative representation method to visualize phylogenic trees in a compact manner that focuses on rendering the weights of the nodes.

## Methods

A treemap [5] is a drawing method that represents a hierarchy as nested rectangles. Each element of the hierarchy (in our case, taxon) is converted to a rectangle. Each sub-element is then a sub-rectangle. Additionally, the area of the rectangle is proportional to the associated quantity (in our case, assigned read number). The final result is a tile-like figure where the larger tiles represent the more abundant species in the dataset. One interesting property is that a sub-branch of the tree is represented as an intermediate rectangle, and the drawing method assures that the area of this intermediate rectangle is proportional to the sum of the reads assigned to the sub-branch. Thus, all the reads are represented in a compact flat view that maintains the tree hierarchy.

In the original treemap algorithm only the leaves are weighted, and the parent nodes are used to group leaves as containers to render the hierarchy. In the case of metagenomic data, some reads may be assigned to the taxon representing the last common ancestor, meaning internal nodes (not leaves) can also have weight. Thus, MetaTreeMap needs to fit with these two paradigms, 1) internal nodes are containers and 2) all reads are represented in rectangular areas.

**Fig 1:**
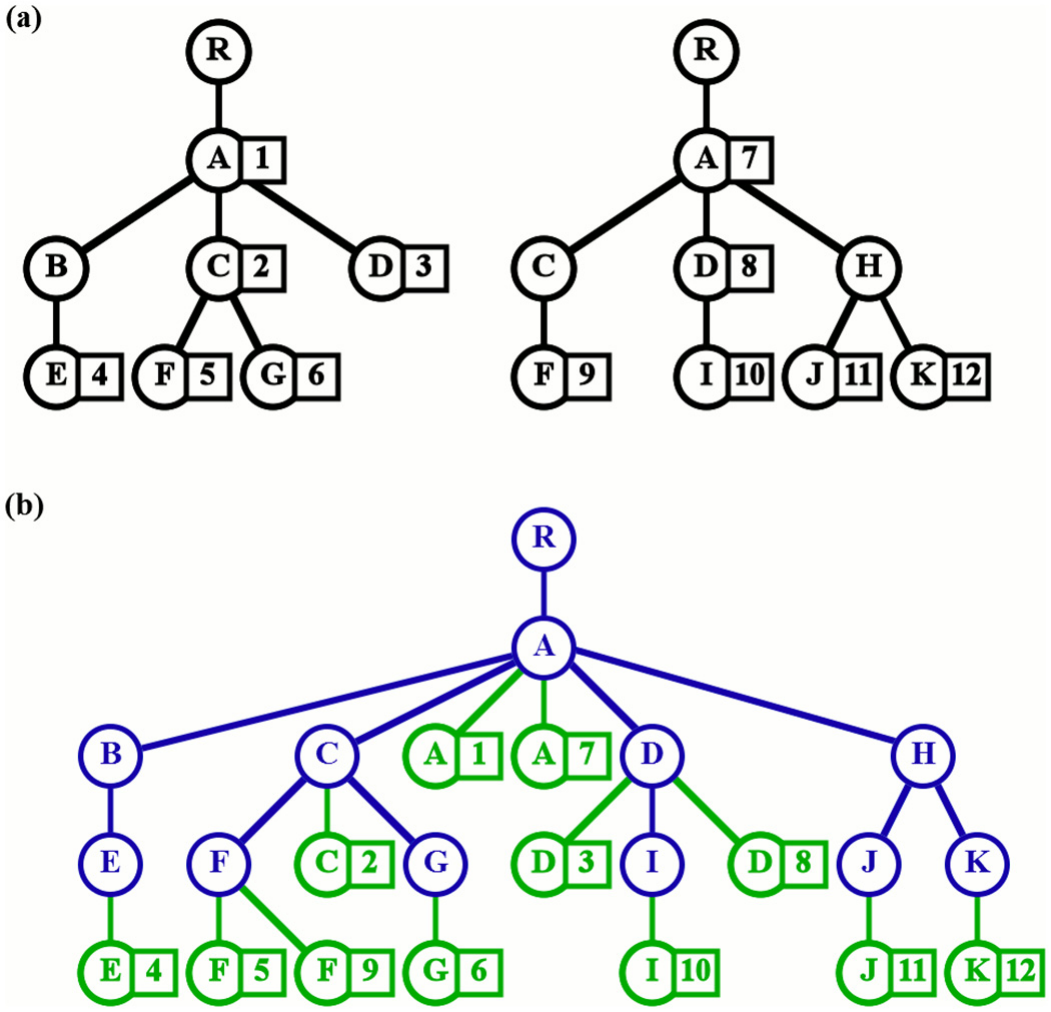
Construction of the MetaTreeMap data structure. (a) Trees representing the two datasets to be compared. (b) Merged tree constructed by the software. Circles are nodes, squares are assigned reads. From the initial datasets (black), MetaTreeMap creates a skeleton tree (blue) with leaves (green).

In our implementation, all the taxa are used to construct a skeleton tree that determines the hierarchy, then the reads are distributed to leaf nodes, children of the associated taxa, and named accordingly (Fig 1) When multiple datasets are compared, a unique skeleton tree is constructed that contains the taxa from all of the datasets, then leaf nodes are created independently for each sample. To simultaneously visualize both hierarchy and quantity, all nodes are drawn as rectangles and leaf areas are calculated according to the associated quantities. Finally, the internal nodes are drawn to encapsulate their children.

To assure that every taxon can be found in the representation, we implemented features such as tooltips and a search field described in the Usage section below. We also display a list of taxa in tabular form that is synchronized with the treemap.

## Results

MetaTreeMap is written in JavaScript. The software uses the treemap layout from the D3 library (https://d3js.org/), then extends its functionalities to fit with metagenomic data specifications. It implements dedicated options (see below) and encapsulates the tool in a graphical user interface using Bootstrap (http://getbootstrap.com/). The final script is distributed as a library (see Discussion) that manages customization, and import and export of data.

### Input and Output

The default input file for MetaTreeMap is a hierarchy of taxon with read numbers assigned to each element. The data should be in JSON format with specific fields that correspond to those of MetaBin (see MetaBin specifications). However, MetaTreeMap includes a module to convert other custom JSON files to the proper input format (in the menu bar see: Import / Convert / Format…). The converter module can also be used to manage simple tabular files that list taxa with scores assigned to each taxon (and NCBI Taxonomy IDs). MetaTreeMap builds the tree based on the current NCBI Taxonomy database (as of 03 May 2016). After conversion, JSON files are exported in MetaTreeMap format.

**Fig 2:**
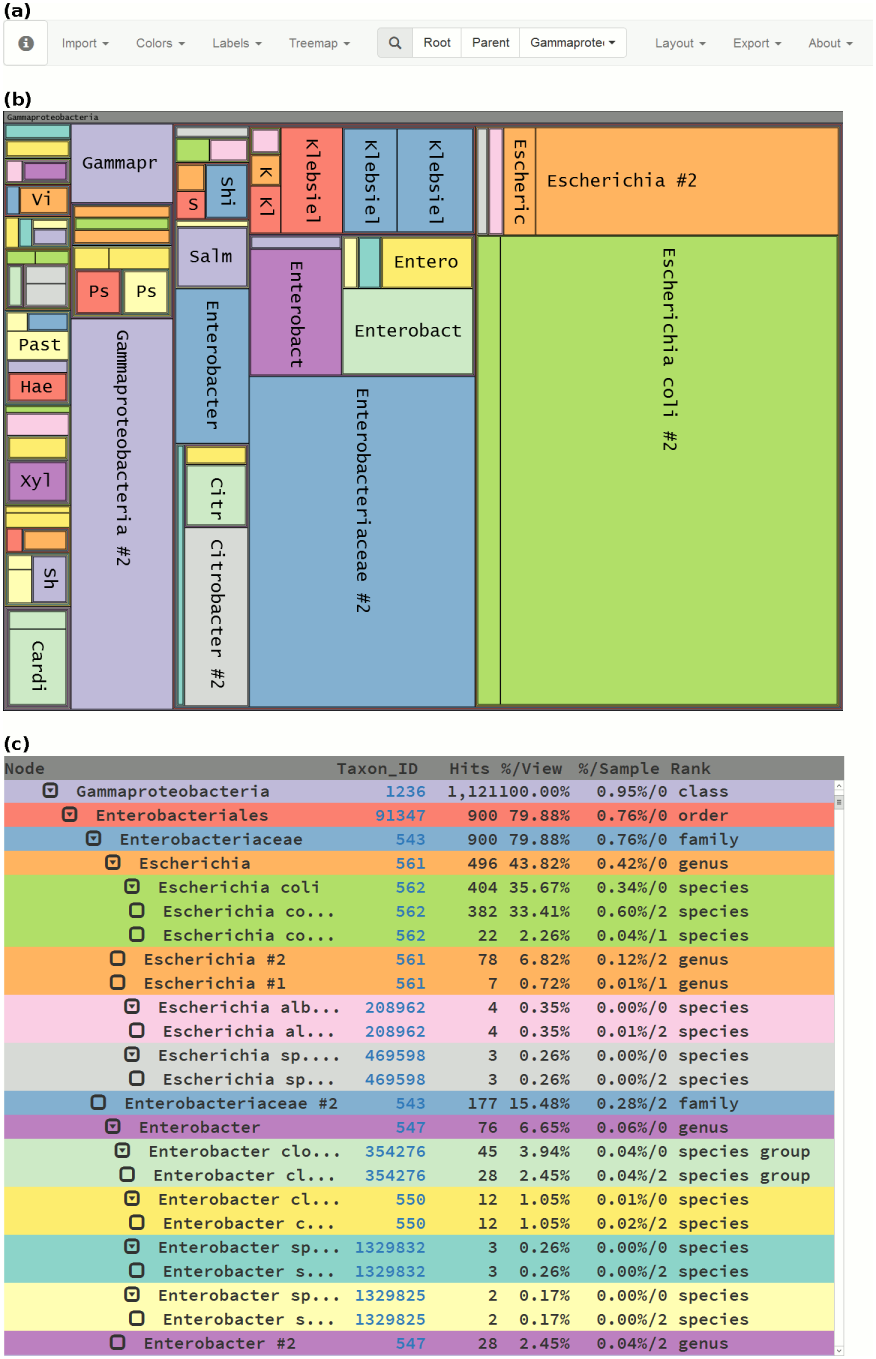
Visualization of Gammaproteobacteria present in two human gut samples. (a) Menu bar. (b) Treemap view of Gammaproteobacteria from samples F1-S and F1-T [6]. All phylogenic ranks are displayed, nodes are colored by taxon name. (c) Synchronized table view of the same data.

In the visualization module, either a single file can be loaded, or multiple files can be loaded simultaneously for comparison. To assure privacy, no data is transmitted to our server, as calculations are performed on the client side. The data are rendered both as a treemap (Fig 2b) and as a synchronized table (Fig 2c). The menu bar (Fig 2a) allows for further customization. The treemap can be saved in either vector graphic (SVG) or bitmap (PNG) format, and the table can be saved as a tabular file (TSV). When multiples files are inputted, a merged version of the tree, where the read counts are summed, can be exported in JSON format.

### Usage

**Fig 3:**
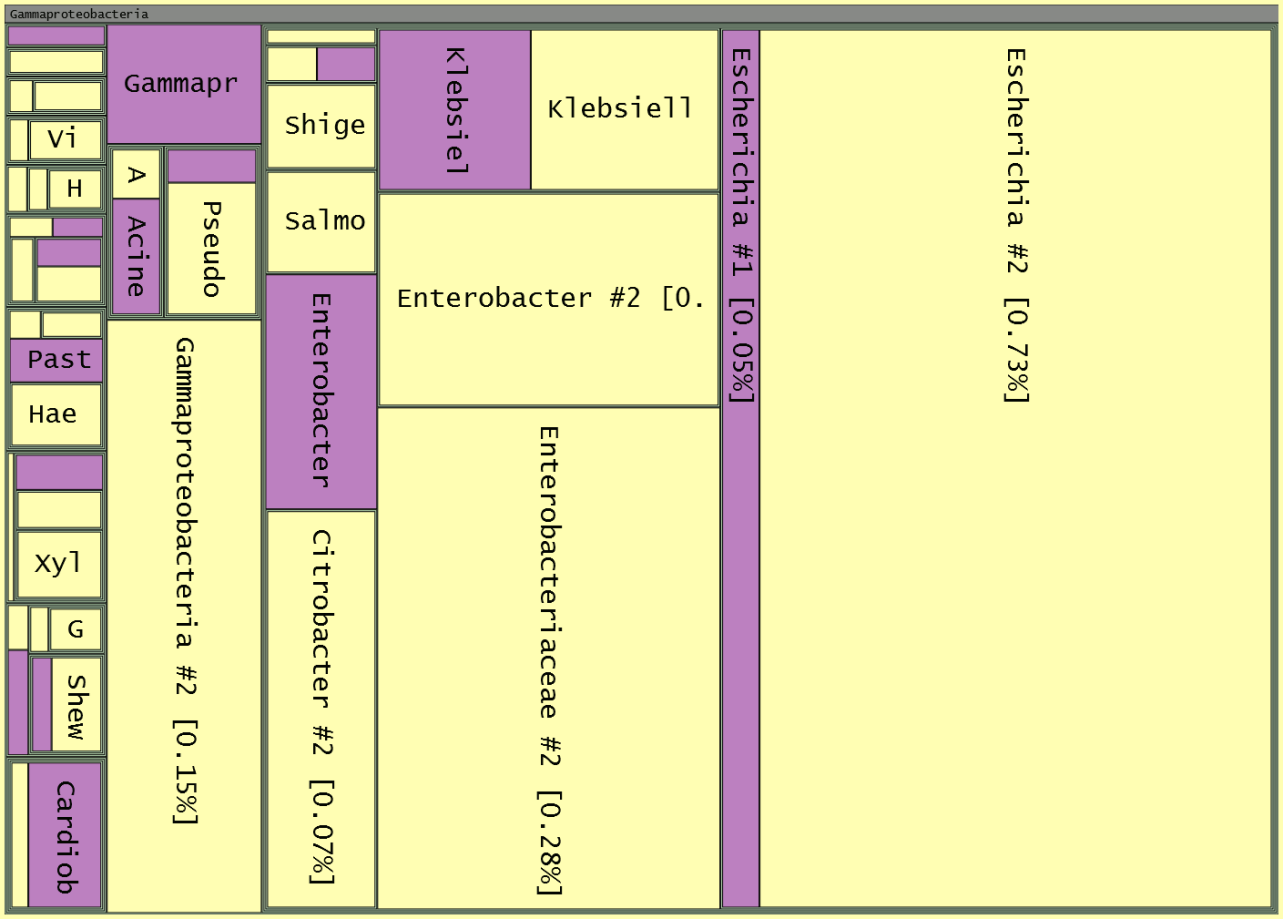
Comparison of Gammaproteobacteria present in two human gut samples. Treemap view of Gammaproteobacteria from samples F1-S and F1-T [6]. Phylogenic rank genus and above are displayed, nodes are colored by sample. The percentage of reads by sample is displayed inside square brackets.

By default, all leaves are labeled and colored according to the taxon they represent. Mousing over a rectangle highlights its sub-branch and displays relevant information in a tooltip (taxon name, taxon ID, rank, number of reads, percentage of reads, and sample number). Users can browse through the hierarchy by simply clicking on a rectangle or on the treemap header to trigger zoom in or zoom out events, respectively. Using the Search field in the menu bar or clicking in the table view allows for quick focus on a taxon of interest. Colors can be changed to distinguish branches by specific phylogenic rank. For convenience, the lines in the table view are colored and synchronized with the treemap view. A cutoff can be selected (see in the menu bar: Treemap / Cutoff) to set the lowest phylogenic rank to be displayed. Descendants of taxa at the specified rank will not be shown (Fig 3). In addition to taxa, labels can also be set to fit with phylogenic rank, customized to display additional information, or hidden, according to user preference. Also, font size, color palette and background color can be changed.

### Multiple samples

Multiple data files can be loaded and visualized in the same treemap. A list of the currently displayed samples is shown by clicking on the “i” button in the menu bar. By default, the treemap displays normalized quantities, with each sample taking up the same amount of space. The areas of the rectangles are proportional to the percentage of reads for each node in its sample. The table module indicates the percentage of each taxa for each sample according to the current view. Optionally, the nodes can be colored according to sample number to simplify the comparison (Fig 3). In addition, normalization can be turned off to directly size the rectangles according to read number (in the menu bar see: Treemap / Proportion By / Hits). The whole treemap can also be divided equally among all leaves to more easily investigate the less abundant species (set Proportion By Taxon).

## Discussion

MetaTreeMap is a JavaScript library that implements a set of modules for visual analysis of metagenomic data. The library is distributed under the Berkeley Software Distribution License Simplified. It can be downloaded freely and included in any web site. The source code is maintained in our laboratory and is open to the community for further development on GitHub (http://github.com/mhebrard/MetaTreeMap/). A web interface is hosted by RIKEN (http://metasystems.riken.jp/visualization/treemap/) that allows for online analysis without registration. The visualization and the analysis is computed on the client side, thus no scientific data is sent or stored on the host server.

The core module constructs phylogenic trees and focuses on taxonomic assignment visualization. It renders an overview of the whole dataset rectangularly, thus optimizing screen space. The view is dynamic, managing zoom events and displaying detailed information by mouse interactions. A second module renders the complete dataset in an interactive table. The results of the analysis can be saved for downstream use, including the configuration state. Both views are highly customizable to fit user preferences. Final figures can be saved in vectorial format or as high resolution images for publication purposes.

The data structure used by MetaTreeMap is created independently of the views. This separation simplifies the implementation of new layouts and the synchronization of interactions and customization for the views. We continue to investigate different patterns of representation to add to MetaTreeMap that could highlight interesting properties of the data and help scientists to comprehend and uncover them faster and more easily.
